# Vaccination accelerates hepatic erythroblastosis induced by blood-stage malaria

**DOI:** 10.1186/s12936-020-3130-2

**Published:** 2020-01-29

**Authors:** Denis Delic, Frank Wunderlich, Saleh Al-Quraishy, Abdel-Azeem S. Abdel-Baki, Mohamed A. Dkhil, Marcos J. Araúzo-Bravo

**Affiliations:** 1 Department of Biology, Heinrich-Heine-University, Duesseldorf, Germany; 2 Boehringer Ingelheim Pharma, Biberach, Germany; 3 Department of Zoology, College of Science, King Saud University, Riyadh, Saudi Arabia; 4 Department of Zoology, Faculty of Science, Beni-Suef University, Beni Suef, Egypt; 5 Department of Zoology and Entomology, Faculty of Science, Helwan University, Cairo, Egypt; 6 Group of Computational Biology and Systems Biomedicine, Biodonostia Health Research Institute, San Sebastián, Spain; 7 Ikerbasque, Basque Foundation for Science, Bilbao, Spain

**Keywords:** Liver, Blood-stage malaria, *Plasmodium chabaudi*, Protective vaccination, Extramedullary erythropoiesis

## Abstract

**Background:**

Vaccination induces survival of otherwise lethal blood-stage infections of the experimental malaria *Plasmodium chabaudi*. Blood-stage malaria induces extramedullary erythropoiesis in the liver. This study investigates how vaccination affects the course of malaria-induced expression of erythrocytic genes in the liver.

**Methods:**

Female Balb/c mice were vaccinated at week 3 and week 1 before challenging with 10^6^ *P. chabaudi*-parasitized erythrocytes. The non-infectious vaccine consisted of erythrocyte ghosts isolated from *P. chabaudi*-infected erythrocytes. Gene expression microarrays and quantitative real-time PCR were used to compare mRNA expression of different erythrocytic genes in the liver of vaccination-protected and non-protected mice during infections on days 0, 1, 4, 8, and 11 *p.i.*

**Results:**

Global transcriptomics analyses reveal vaccination-induced modifications of malaria-induced increases in hepatic gene expression on days 4 and 11 *p.i.* On these days, vaccination also alters hepatic expression of the erythropoiesis-involved genes *Ermap, Kel, Rhd*, *Rhag*, *Slc4a1, Gypa, Add2, Ank1, Epb4.1, Epb4.2, Epb4.9, Spta1, Sptb, Tmod1*, *Ahsp, Acyp1*, *Gata1, Gfi1b, Tal1, Klf1, Epor*, and *Cldn13*. In vaccination-protected mice, expression of these genes, except *Epb4.1*, is significantly higher on day 4 *p.i.* than in un-protected non-vaccinated mice, reaches maximal expression at peak parasitaemia on day 8 *p.i.,* and is slowed down or even decreased towards the end of crisis phase on day 11 *p.i..* After day 1 *p.i., Epor* expression takes about the same course as that of the other erythroid genes. Hepatic expression of *Epo,* however, is delayed in both vaccinated and non-vaccinated mice for the first 4 days *p.i.* and is maximal at significantly higher levels in vaccinated mice on day 8 *p.i.*, before declining towards the end of crisis phase on day 11 *p.i.*

**Conclusion:**

The present data indicate that vaccination accelerates malaria-induced erythroblastosis in the liver for 1–2 days. This may contribute to earlier replenishment of peripheral red blood cells by liver-derived reticulocytes, which may favour final survival of otherwise lethal blood-stage malaria, since reticulocytes are not preferred as host cells by *P. chabaudi*.

## Background

Malaria has caused about 219 million cases and about 435,000 deaths globally in 2017, with about 266,000 deaths in children aged under 5 years [1]. Currently, promising vaccine candidates are tested, but an effective anti-malarial vaccine is not yet commercially available [2–4].

The malaria-causing agents are parasitic protozoans of the genus *Plasmodium*, whose blood-stages, developing and multiplicating within host erythrocytes, exclusively cause morbidity and mortality of malaria [3]. The deadliest human malaria species is *Plasmodium falciparum* causing about 99% of global malaria-related deaths in humans [1]. *Plasmodium falciparum* shares several characteristics with *Plasmodium chabaudi* in mice which is, therefore, widely used as an appropriate experimental system to study host defense mechanisms against blood-stage malaria [5, 6]. The *P. chabaudi* model is also appropriate to study effects of vaccination on the outcome of blood-stage malaria. Using an experimental non-infectious vaccine, survival of mice can be raised from 0% to over 80% [7, 8]. This vaccine induces a healing course of otherwise lethal infections. For instance, primary infections with 10^6^ *P. chabaudi*-parasitized erythrocytes take a similar course in terms of parasitaemia in vaccinated and non-vaccinated mice of the inbred mouse strain Balb/c [8]. The prepatent phase lasts for about 3 days, before the patent period begins on day 4 *post infectionem* (*p.i.*), characterized by the appearance of parasitized erythrocytes in the peripheral blood. The parasitaemias vary among infected mice between 1–5% without any difference between vaccinated and non-vaccinated mice. Maximum parasitaemia is reached on day 8 *p.i.* with about 60% parasitaemia in non-vaccinated mice, whereas it is reduced to about 40% in vaccinated mice. Peak parasitaemia is followed by the crisis phase characterized by dramatically declining parasitaemias to 5–1% within 3–4 days. All non-vaccinated mice succumb to infection during crisis, whereas over 80% of the vaccinated mice survive the infections [8].

The massive clearance of *P. chabaudi*-parasitized erythrocytes from circulation during crisis is ascribed to the spleen, which is widely considered as the exclusive effector organ against blood-stage malaria [9, 10]. Indeed, the spleen is thought of using the same mechanisms for eliminating malaria-parasitized erythrocytes in its red pulp, by which it normally removes senescent erythrocytes from circulation. Moreover, the liver is increasingly recognized as an effector organ against blood-stage malaria [11] due to its intrinsic immune system, its specific iron disposal system, and its capability to remove senescent and other aberrant erythrocytes including *P. chabaudi*-parasitized erythrocytes [11–16].

In *P. chabaudi* infections, the dramatic low levels of peripheral erythrocytes towards the end of crisis occur, though erythropoiesis is concomitantly active evidenced as increasing levels of peripheral reticulocytes [8]. Erythropoiesis is a highly complex process proceeding from the commitment of a haematopoietic stem cell to the megakaryocyte-erythroid lineage, which generates the primitive progenitors of the erythrocyte lineage, i.e., the slowly proliferating burst-forming unit-erythroid cells followed by the rapidly expanding colony-forming unit-erythroid (CFU-e) cells. These in turn are the precursors for the nucleated pro-erythroblasts and erythroblasts [17–19], which are arranged in erythroblastic islands organized around a central macrophage [20, 21]. Erythroblasts denucleate to reticulocytes which terminally differentiate to erythrocytes [20, 22]. Erythropoietin (EPO) and erythropoietin receptor (EPOR), respectively, play a critical role in erythropoiesis, in particular during erythroblastosis. EPOR is constitutively expressed on CFU-e and erythroblasts [23, 24]. EPO-sensitive erythropoiesis is obviously also critical for the outcome of *P. chabaudi* blood-stage malaria [25, 26]. In particular, EPO-induced proliferation of erythroid precursors has been described to be suppressed by parasite factors thus causing severe anaemia and exacerbating effects on the final outcome of *P. chabaudi* infections [27].

Erythropoiesis takes place in the bone marrow [20, 21]. Evidence, however, is increasing that, under specific stress situations, erythropoiesis can also take place outside the bone marrow. This stress or extramedullary erythropoiesis has been reported to occur in several organs, particularly in the spleen, of mice and even humans [20, 28–31]. Previously, the spleen was found to be a site of increased erythropoiesis in *P. chabaudi*-resistant C57BL/6 mice [32]. Thereafter, the liver was also found to be a site of malaria-induced erythropoiesis. Thus, blood-stage infections with the non-lethal *Plasmodium yoelii* 17XL in C57BL/6 mice were reported to induce large cell clusters containing TER119^+^ nucleated reticulocytes in the parenchymal space of the liver [33, 34]. This aspect has been neglected until recently when several erythrocytic genes were detected by transcriptomics screening analyses to be expressed in the liver of mice infected with *P. chabaudi* [35, 36]. At present, however, there is only poor information available on the dynamics of stress-induced extramedullary erythropoiesis in general and, in particular, on the dynamics of malaria-induced extramedullary erythropoiesis in the liver at the gene expression level. To get a better understanding of extramedullary erythropoiesis in the liver induced by blood-stage malaria and, particularly, its modulation by vaccination, this study comparatively analyses the time-course of expression of a series of erythrocytic genes in the liver during primary blood-stage infections of *P. chabaudi* taking a lethal outcome in non-vaccinated mice and, in parallel, during healing infections in vaccination-protected surviving mice. These data are discussed with respect to *P. chabaudi*-induced changes in peripheral blood previously analysed under identical experimental conditions as used here [8].

## Methods

### Mice

Experiments were performed with female Balb/c mice aged 10–12 weeks, which were delivered from the central animal facility of the University of Düsseldorf, where mice were bred under specified pathogen-free conditions. During infection experiments, mice were housed in plastic cages and received a standard diet (Woehrlin, Bad Salzuflen, Germany) and water ad libitum.

### Protective vaccination

Vaccination was performed under identical experimental conditions as described previously [8]. The non-infectious vaccine, consisting of erythrocyte ghosts isolated from *P. chabaudi*-parasitized erythrocytes, was prepared as detailed previously [7, 8, 37]. These membrane ghosts were previously characterized to contain parasite-synthesized proteins [38, 39]. Approximately 10^6^ ghosts suspended in 100 µl Freund’s complete adjuvant (FCA) were subcutaneously injected at weeks 3 and 1 before infection with *P. chabaudi* blood-stage malaria. In parallel, only FCA was injected in control mice.

### Blood-stage malaria of *P. chabaudi*

*Plasmodium chabaudi* infections were maintained in outbred mice by weekly passages of infected blood under sterile conditions. The non-clonal line of *P. chabaudi* [40], resembles *P. chabaudi* AS clone in terms of restriction fragment length polymorphism as well as dihydrofolate reductase and cysteine protease sequence identities [41]. Moreover, the used line of *P. chabaudi* is self-healing as the AS clone, which is under control of genes of the H-2 complex and the non-H-2 background as well as sex and sex hormones of the infected mouse strain [42]. The Balb/c mice were infected with 10^6^ *P. chabaudi*-infected erythrocytes. Parasitaemia was evaluated in Giemsa-stained smears from tail blood and erythrocytes were counted in a Neubauer chamber as described previously [8]. Both groups of vaccinated and non-vaccinated mice contained 4 ‘control’ mice, which were not sacrificed for liver sampling. All 4 mice in the non-vaccinated group succumbed to infection during crisis. In the vaccinated group, however, only 1 mouse succumbed to infection during crisis, whereas 3 mice survived the infection for at least 3 weeks, in accordance with previous results [8].

### Liver sampling

To analyse hepatic gene expression during the course of *P. chabaudi* infections, both vaccinated (V) and non-vaccinated mice (N) were concomitantly infected, and groups of 3 mice were sacrificed at different time points of infections: upon infection on day 0 *p.i.* (groups Vd0 and Nd0), at early prepatency on day 1 *p.i.* (groups Vd1 and Nd1), at early patency on day 4 *p.i.* (groups Vd4 and Nd4) when parasitized erythrocytes begin to appear in peripheral blood with parasitaemias varying between 1–5%, at peak parasitaemia on day 8 *p.i.* (groups Vd8 and Nd8), and towards the end of the crisis phase on day 11 *p.i.* (groups Vd11 and Nd11) when parasitaemia declined to 5–1%. The course of parasitaemia was previously determined in mice sacrificed in the different groups [43], which corresponded to that in living infected mice under identical experimental conditions [8]. Livers were aseptically removed from sacrificed mice, rapidly frozen in liquid nitrogen and stored at − 80 °C until use.

### RNA isolation

Frozen livers were individually ground in a mortar under liquid nitrogen. Total RNA was isolated from ‘pulverized’ aliquots of each individual liver by the standard Trizol protocol (Qiagen, Hilden, Germany), followed by an additional cleaning up with the miRNeasy Kit (Qiagen). The Agilent 2100 Bioanalyser platform (Agilent Technologies) was then used to check integrity and quality of RNA. The RIN values of all 30 liver RNA samples ranged between 8.7 and 9.1.

### Cy3 labelling of RNA

Each RNA sample (equivalents of 100 ng) were used to produce Cy3-labeled cRNA using the Agilent Low Input Quick Amp Labeling Kit (Agilent Technologies) according to the manufacturer’s protocol. Yields of cRNA and dye-incorporation were determined with the ND-1000 Spectrophotometer (NanoDrop Technologies). The incorporations ranged between 18 and 23 fmol Cy3/ng cRNA.

### Hybridization of Agilent mouse whole genome oligo microarrays

Agilent’s 8x60 K oligo microarrays (design number 028005) were used, which contained 8 arrays per slide. Each array displayed 39,430 Entrez Gene RNAs. Using the Agilent Gene Expression Hybridization Kit, hybridization was performed according to the Agilent processing protocol (Agilent technologies). Specifically, 600 ng Cy3-labelled fragmented cRNA was hybridized overnight at 65 °C using Agilent’s recommended hybridization chamber and oven. Finally, the microarrays were washed with the Agilent Gene Expression Wash Buffer 1 for 1 min at room temperature and then with the preheated Agilent Gene Expression Wash Buffer 2 at 37 °C for 1 min.

### Scanning and analyses of microarrays

Microarrays were scanned with the Agilent’s Microarray Scanner System (Agilent Technologies). Microarray image files were processed with the Agilent Feature Extraction Software determining feature intensities (including background substraction), rejecting outliers and calculating statistical confidences of the array spots. All the 30 microarrays were normalized by the quantile method. When required, the expression variance was stabilized through the log_2_ transform. In-home functions developed in Matlab (MathWorks) were used to construct heat maps, the hierarchical clustering dendrograms (calculated using the unweighted pair group method with arithmetic mean and Euclidean distance measure), and the principal component analysis (PCA). The microarray data have been deposited at the NCBI’s Gene Expression Omnibus (GEO) database with accession number GSE129133.

### Microarray-based analyses of genes involved in erythropoiesis

The used Agilent’s 8x60K microarrays contain probes for the following genes: *Acyp1* (*acylphosphatase 1 erythrocyte isozyme*), *Add2* (*adducin2*), *Ahsp* (*α hemoglobin stabilizing protein*), *Ank1* (*ankyrin1*), *Cldn13* (*claudin13*), *Epb4.1* (*Erythrocyte membrane protein band 4.1*), *Epb4.2* (*Erythrocyte membrane protein 4.2*), *Epb4.9* (*Erythrocyte membrane protein 4.9*), *Epo* (*erythropoietin*), *Epor* (*erythropoietin receptor*), *Ermap* (*erythroblast membrane associated protein*), *Gata1* (*GATA*-*binding factor 1*), *Gfi1b* (*growth factor independent 1B transcriptional repressor*), *Gypa* (*glycophorin A*), *Kel* (*Kell blood group antigen)*, *Klf1* (*Krueppel*-*like factor 1*), *Rhag* (*Rh*-*associated glycoprotein*), *Rhd* (*Rh blood group D antigen*), *Slc4a1* (*solute carrier family 4 member 1 *=* protein band 3*), *Spta1* (*spectrin α erythrocytic 1*), *Sptb* (*Spectrin β erythrocytic*), *Tal1* (*T*-*cell acute lymphocytic leukemia protein 1*), and *Tmod1* (*tropomodulin 1*). The expression profiles of these 23 genes were determined from the normalized microarrays prepared from the individual livers of both vaccinated and non-vaccinated mice during infections with *P. chabaudi* on days 0, 1, 4, 8, and 11 *p.i.* Gene expression levels, measured as light intensities above the normalization level, were given as mean ± SD as a dispersion metric in all figures. T-test was used to determine statistical significance of differences of gene expression levels between vaccinated and non-vaccinated mice both at a given day *p.i.* and during the intervals between the different sampling days *p.i.* The number of ‘*’ marks in the figures that appear over interval lines between sampling points or over the sampling points are the number of zeros after the decimal point of the *p*-values for the statistical significance of the difference in instant of the two series and of the difference between two intervals, respectively.

### Quantitative real-time PCR of erythroid genes

High Capacity cDNA Reverse Transcription Kit (Life Technologies) and TaqMan mRNA assays (Life Technologies) were used to perform reverse transcription of mRNAs coding for the following proteins: *RHD* (assay ID: Mm00456910_m1), *ERMAP* (Mm_00469273_m1), *GATA1* (Mm01352636_m1), *SLC4A1* (Mm00441492_m1), *CLDN13* (Mm00491038_s1), *ADD2* (Mm00478923_m1), *ACYP1* (Mm00481325_m1), *EPB4.2* (Mm00469111_m1), *EPB4.9* (Mm00469121_m1), *EPOR* (Mm01202755_m1), and *EPO* (Mm00833882_m1). The TaqMan^®^ gene expression master mix (Life Technologies) was used for PCR reactions according to the instructions given by the manufacturer on a 7900HT real-time PCR System, as previously described [35]. Raw Ct values were calculated using the SDS software v.2.4 with *GAPDH* (Glycerinaldehyd-3-phosphat-Dehydrogenase) for normalization, and the comparative Ct method (2^−ΔΔCt^) was used to calculate fold change of expression [44]. Statistical significance of corresponding data sets between vaccinated and non-vaccinated mice were analysed with the two-tailed unpaired heteroskedastic Student´s T-test (* = *p*-value < 0.05).

## Results

### Global transcriptomics analyses reveal modifications by vaccination of malaria-induced hepatic gene expression on days 4 and 11 *p.i*

The effect of protective vaccination on the time course of erythroid gene expression in the liver induced by *P. chabaudi* blood-stage malaria was analysed using mouse whole genome 8x60K oligo microarrays. In toto, 30 microarrays were used for 30 livers prepared from the 10 different groups of vaccination-protected and un-protected non-vaccinated mice at the different phases of infections, i.e., upon infection on day 0 *p.i.*, at early prepatency on day 1 *p.i.,* at early patency on day 4 *p.i.*, at peak parasitaemia on day 8 *p.i.*, and towards the end of crisis on day 11 *p.i.*

**Fig. 1:**
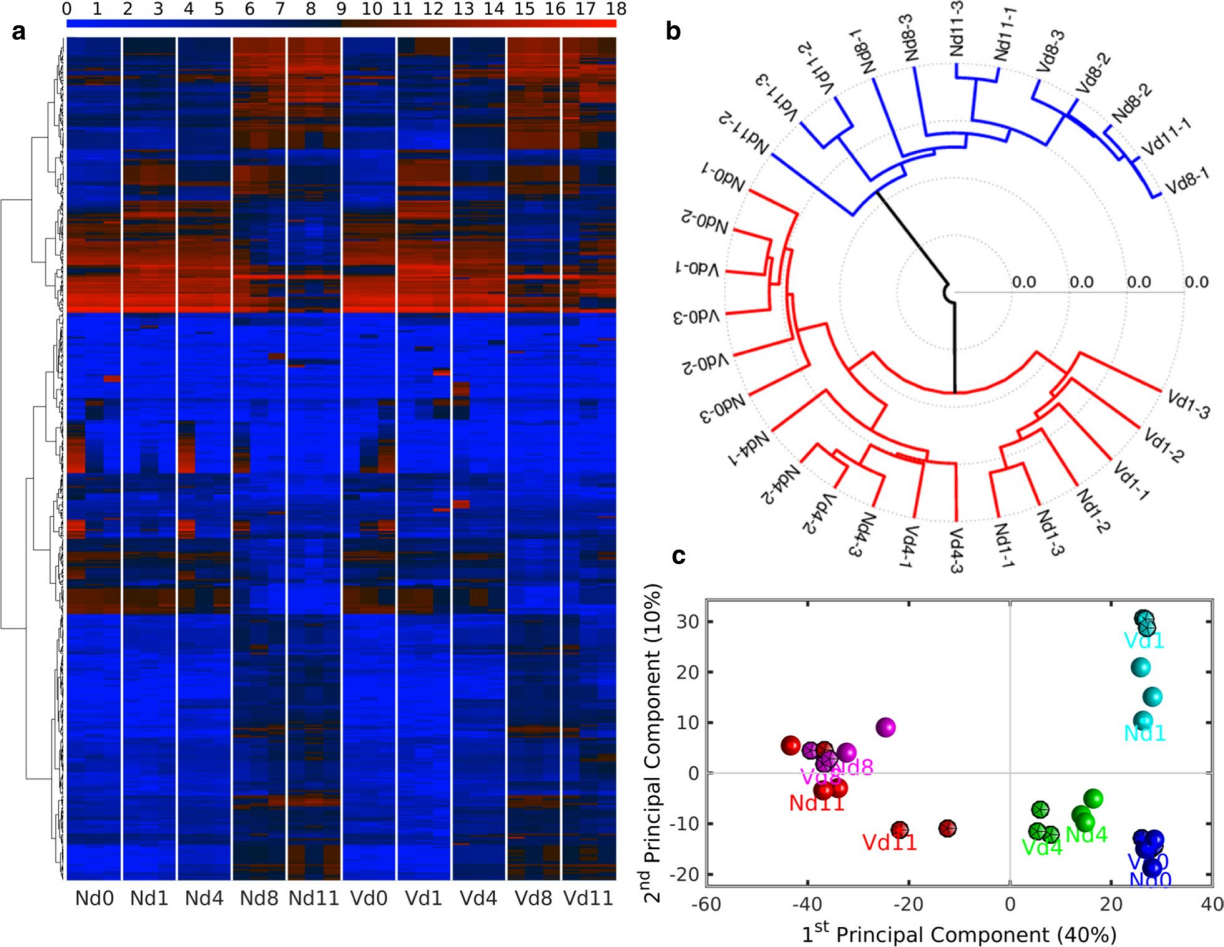
Global expression profile of mRNAs in the liver of non-vaccinated (N) and vaccination-protected mice (V). **a** RNA was isolated from individual livers prepared on different days (d0, d1, d4, d8, d11) during infection with *P. chabaudi* blood-stage malaria. Gene expression levels for the individual livers were determined by microarrays and hierarchically clustered. Log_2_ transformed expression levels range from 0 to 18 as indicated at the top with blue (low expression) and red (high expression), respectively. **b** Hierarchical clustering of liver samples prepared from vaccination-protected (V) and non-vaccinated mice (N) during different phases of infections with *P. chabaudi* malaria on the indicated days (d0, d1, d4, d8, d11). Suffices indicate the three different livers taken per time point. The correlation metric and the average linkage method were used to construct the dendogram. **c** Principal Component Analysis (PCA) of gene expression data. The 1st principal component (PC1) captures 40% of the gene expression variability and the 2nd PC2 captures 10% of the variability. The Nd0 populations are depicted by blue spheres, the Nd1 by cyan spheres, the Nd4 by green spheres, the Nd8 by magenta spheres, the Nd11 by red spheres, the Vd0 by blue icosahedra, the Vd1 by cyan icosahedra, the Vd4 by green icosahedra, the Vd8 by magenta icosahedra and the Vd11 by red icosahedra

Figure 1a shows the heatmap of gene expression profiles of those probes whose range or variation across all samples were at least 7 in the 10 different groups. In general, there is a good replicability among the three replicates of each analysed condition. The heatmap also shows a gradual change in gene expression across time both in non-vaccinated and vaccinated samples with an abrupt change in gene expression at peak parasitaemia on day 8 *p.i.* As the heatmap, the hierarchical clustering of the 30 different individual samples, shown as a dendogram in Fig. 1b, also reveals a good replicability among the three replicates of each analysed condition and the abrupt change in gene expression on day 8 *p.i.* There are two main branches splitting the samples taken on days 0, 1, and 4 *p.i.* (red branches in Fig. 1b) from the samples taken on days 8 and 11 *p.i.* (blue branches in Fig. 1b). The principal component analysis (PCA) of gene expression (Fig. 1c) shows that the 1st principal component (PC1) accounts for 40% of the gene expression variability and the 2nd PC2 captures 10% of the variability. The 1st principal axis, that is the most informative, separates between those liver samples taken before day 8 *p.i.* (positive coordinates) and those taken after day 8 *p.i.* samples (negative coordinates).

Collectively, the global transcriptomics analysis results indicate (i) that constitutive expression of the vast majority of hepatic genes is not essentially changed by protective vaccination, (ii) that *P. chabaudi* infections induce changes in hepatic RNA expression, being usually highest at peak parasitaemia on day 8 *p.i.* and towards the end of crisis on day 11 *p.i.*, and (iii) that the malaria-induced changes in hepatic gene expression are modified by protective vaccination, particularly at early patency on day 4 *p.i.* and towards the end of crisis on day 11 *p.i.*

### Erythropoiesis-involved genes are expressed in the liver of non-infected mice

**Fig. 2:**
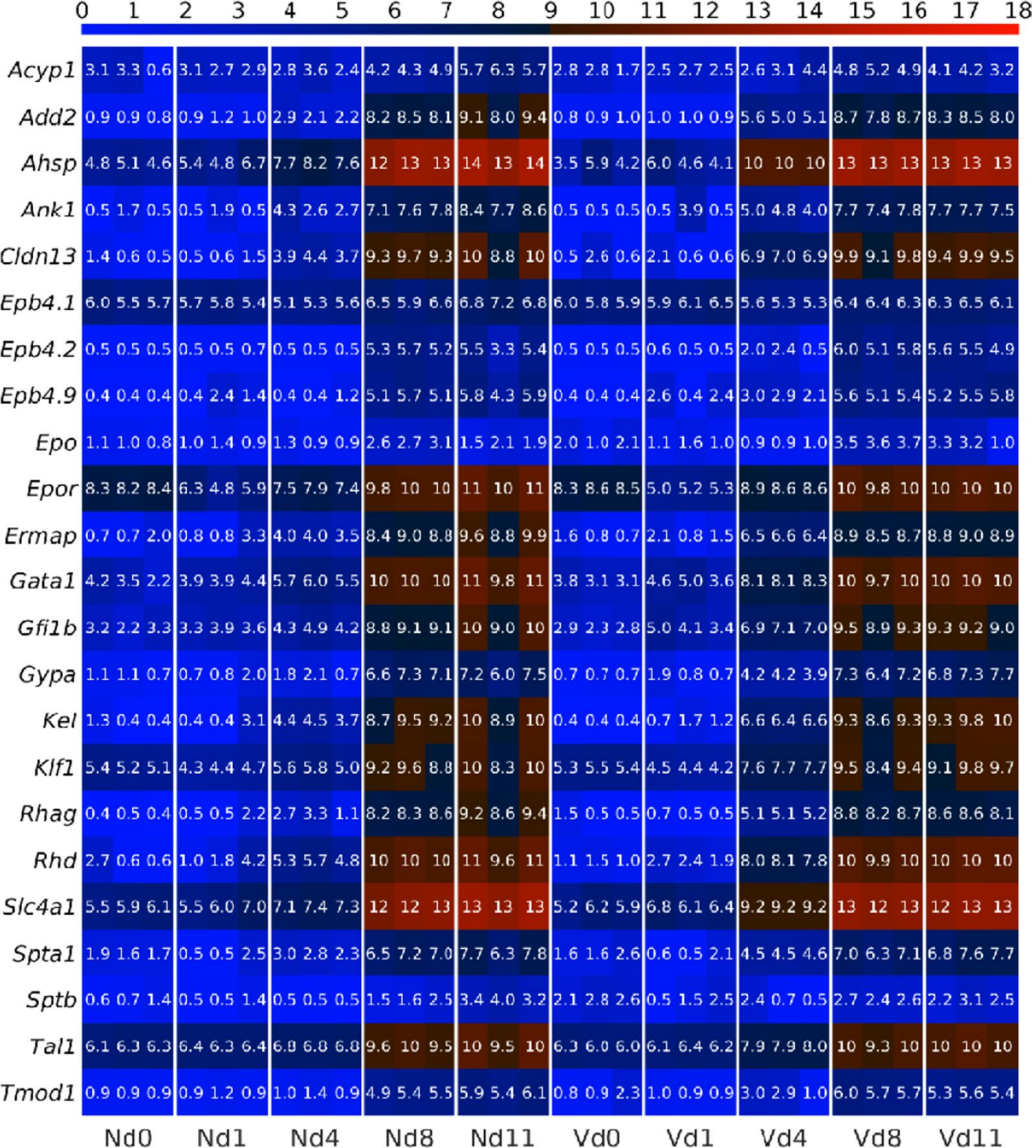
Heatmap of hepatic expression profiles of erythropoiesis-involved genes. RNA was isolated from the individual livers prepared from non-vaccinated (N) and vaccinated (V) mice infected with *P. chabaudi* on days 0, 1, 4, 8, and 11 *p.i.* Log_2_ transformed expression levels range from 0 to 18; the higher is the expression the more red is the colour

The heatmap in Fig. 2 shows the expression profiles of the selected 23 genes known to encode important non-haemoglobin constituents of erythrocytes and the EPO encoding gene known to stimulate erythropoiesis. The numbers in the heatmap indicate the probe-detected expression levels of genes which were log_2_ transformed for variance stabilization. In the liver of non-vaccinated mice on day 0 *p.i.*, the expression of a given gene is similar among the 3 microarrays prepared from the 3 mice (Nd0 in Fig. 2) and also with the corresponding probes on the microarrays of the 3 vaccinated mice on day 0 *p.i.* (Vd0 in Fig. 2). However, the 23 genes reveal varying constitutive expression levels, which, however, are approximately the same at both Nd0 and Vd0. For instance, low constitutive expression levels reveal the genes *Epb4.2*, *Epb4.9*, *Rhag, Ermap*, *Spta*, and *Sptb* at both Nd0 and Vd0, whereas higher expression levels are found for the genes *Epor*, *Slc4a1*, *Gata1*, *Klf1,* and *Tal1* in both vaccinated and non-vaccinated mice (Fig. 2). This supports the view that constitutive expression of erythroid genes in non-infected mice is not essentially affected by vaccination.

### Erythroid genes respond to infections with increasing expressions in non-vaccinated mice

**Fig. 3:**
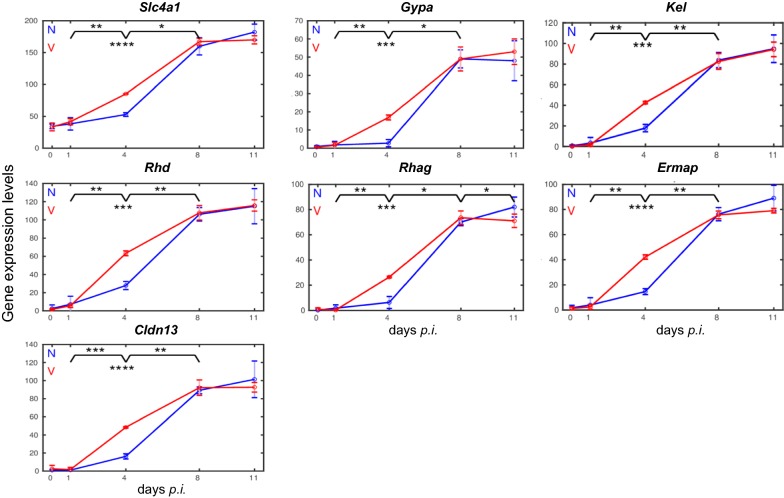
Time courses of hepatic expression of erythroid genes encoding proteins associated with the membrane and extracellular surface of erythroid cells. RNA was isolated from individual livers prepared from vaccination-protected (V, red) and non-vaccinated mice (N, blue) infected with *P. chabaudi* blood-stage malaria on days 0, 1, 4, 8, and 11 *p.i*. Data are plotted in linear scale. Means of 3 microarrays are given ± SD. The number of ‘*’ marks that appear over interval lines between sampling points and over the sampling points are the number of zeros after the decimal point of the *p*-values for the statistical significance of the difference in instant of the two series and of the difference between two intervals, respectively

**Fig. 4:**
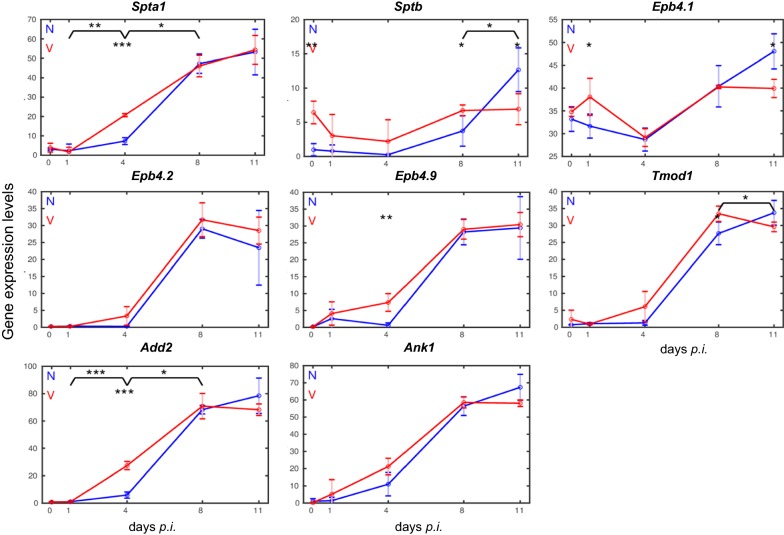
Expression trajectories of erythroid genes coding for proteins associated with the inner surface of the erythroid plasma membrane in the liver of vaccinated and non-vaccinated mice. RNA was isolated from individual livers prepared from vaccination-protected (V, red) and non-vaccinated mice (N, blue) infected with *P. chabaudi* blood-stage malaria on days 0, 1, 4, 8, and 11 *p.i.* Data are plotted in linear scale. Means of 3 microarrays are given + SD. The number of ‘*’ marks indicate significances as detailed in legend to Fig. 3

**Fig. 5:**
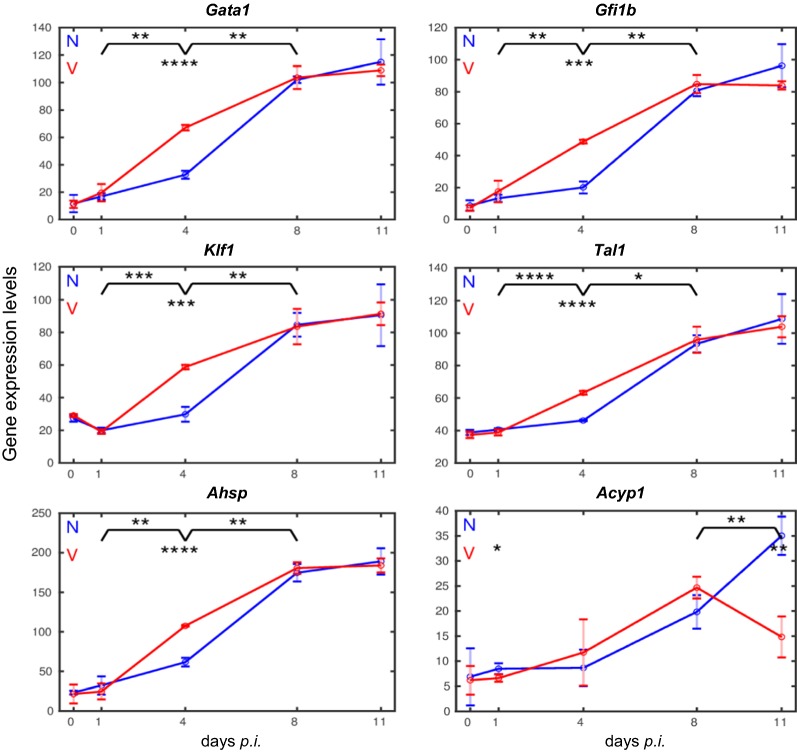
Expression trajectories of genes encoding erythropoiesis-involved transcription factors and other internal erythroid proteins. RNA was isolated from individual livers prepared from vaccination-protected (V, red) and non-vaccinated mice (N, blue) infected with *P. chabaudi* blood-stage malaria on days 0, 1, 4, 8, and 11 *p.i.* Data are plotted in linear scale. Means of 3 microarrays are given ± SD. The number of ‘*’ marks indicate significances as detailed in legend to Fig. 3

**Fig. 6:**
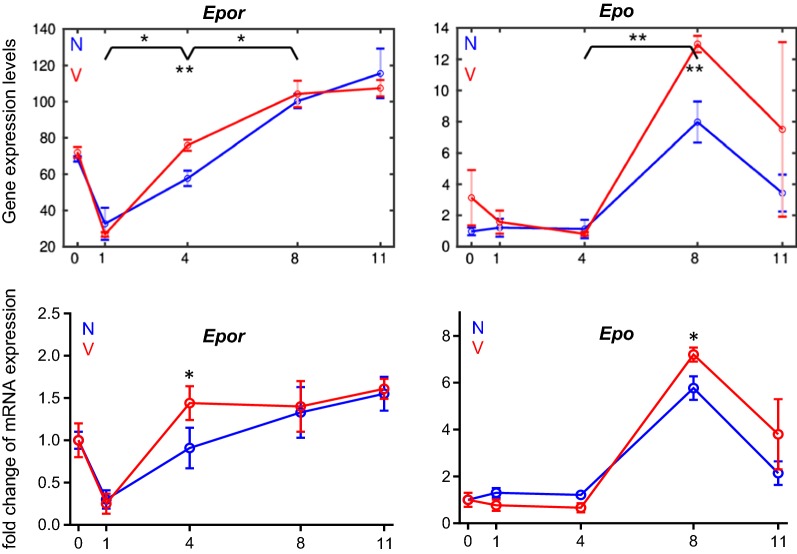
Time course of expression of *Epor* and *Epo* in the liver of vaccination-protected and non-protected mice. RNA was isolated from individual livers prepared from vaccination-protected (V, red) and non-vaccinated mice (N, blue) infected with *P. chabaudi* blood-stage malaria on days 0, 1, 4, 8, and 11 *p.i.* The upper curves show microarray data, the lower curves qPCR data. The number of ‘*’ marks in microarray data indicate significances as detailed in legend to Fig. 3. ‘*’ in qPCR data indicate significant differences between vaccinated and non-vaccinated mice (p < 0.05)

The heatmap in Fig. 2 also shows that blood-stage infections of *P. chabaudi* malaria induce increasing expressions of the 23 erythroid genes in the liver, but with differences among vaccinated and non-vaccinated mice. To make these differences clearer and better comparable, the expression profiles were plotted in a linear scale as time series with the corresponding trajectories presented in Figs. 3, 4, 5, 6.

Blood-stage infections with *P. chabaudi* induce only a slight increase in the expression of all the 23 erythropoiesis-involved genes between days 0 *p.i.* and 4 *p.i.* Thereafter, an abrupt increase in expression is observed at peak parasitaemia on day 8 *p.i.,* and the increase even continues reaching still higher mRNA expression levels towards the end of the crisis phase on day 11 *p.i.* Among genes encoding membrane-associated proteins [45, 46], the gene *Slc4a1* coding for the major integral multi-pass membrane protein band 3 and the gene *Gypa* encoding the major one-pass membrane protein of the *Gyp* family reach maximal mRNA expression levels of approximately 180 and 50 above the normalization level, respectively (Fig. 3). The genes *Kel* and *Rhd* coding for blood groups and *Rhag* coding for the Rh-blood group associated transmembrane glycoprotein reveal maximal expressions of approximately 100, 120, and 90, respectively, on day 11 *p.i.* (Fig. 3). *Ermap* known to encode a protein associated with the outer surface of erythroblasts displays maximal expression of approximately 90 on day 11 *p.i.* A very similar time-course of expression with a maximum level of approximately 100 reveals *Cldn13* (Fig. 3), whose encoded protein has been previously suggested to be associated with erythroblastic islands [35]. Lower maximal expressions on day 11 *p.i.* exhibit those genes, whose encoded proteins are known to be associated with the cytoskeletal meshwork on the inner surface of the erythrocyte plasma membrane (Fig. 4). Among them are *Spta*, *Sptb*, *Epb4.1*, *Epb4.2*, *Epb4.9*, and *Tmod1* [45, 46], whose maximal expressed mRNA levels vary between 15 and 50 above the normalization level, respectively. Only the genes *Add2* involved in the assembly of the spectrin–actin cytoskeletal network and *Ank1* involved in the linkage of integral membrane proteins to the underlying cytoskeletal meshwork display higher expression levels between 80 and 70, respectively (Fig. 4).

The genes *Gata1*, *Gfi1b*, *Klf1*, and *Tal1* encode transcription factors which are known to be critically involved in erythropoiesis [47–49]. These genes also reach high expressions between approximately 80 and 110 above the normalization level towards the end of the crisis phase (Fig. 5). Among all erythroid genes responding to malaria, *Ahsp*, encoding the α haemoglobin stabilizing protein, exhibit the highest mRNA expression of approximately 190 towards the end of the crisis phase (Fig. 5). However, a maximal expression level of only approximately 35 reveals the gene *Acyp1* encoding the erythrocytic acylphosphatase.

Erythropoiesis, in particular erythroblastosis, is critically dependent on EPO signaling through its receptor EPOR [23, 24]. *Epor* and *Epo* display a time-course of mRNA expression, which differs from that determined for the other erythroid genes in two aspects (Fig. 6). *Epor* reveals a sharp decline in its relative expression level from about 75 on day 0 *p.i.* to about 25 on day 1 *p.i..,* before it continuously increases almost linearly reaching its maximal level of about 120 towards the end of crisis on day 11 *p.i.* By contrast, *Epo* expression is impaired during the first 4 days of infection, then it sharply increases reaching its maximum expression of approximately 8 at peak parasitaemia on day 8 *p.i.,* before declining to a level of about 3 towards the end of the crisis phase on day 11 *p.i.*

### Expression of erythroid genes is accelerated in the liver of vaccination-protected mice

In contrast to un-protected non-vaccinated mice, the expression of the vast majority of erythroid genes in the liver of vaccination-protected mice is accelerated during infections, as evidenced by three facts. First, the erythroid genes are significantly much higher expressed at early patency on day 4 *p.i.* than the corresponding genes in the liver of non-vaccinated mice (cf. Figs. 3, 4, 5, 6). This significant higher expression is confirmed when the corresponding genes are directly compared between vaccinated and non-vaccinated mice on day 4 *p.i.* or when the increases in expression levels between day 1 *p.i.* and day 4 *p.i.* is compared in vaccinated mice *vs* non-vaccinated mice. The only exception is *Epb4.1* (Fig. 4): its expression is significantly higher on day 1 *p.i.* in vaccinated mice, then it decreases to approximately the same low level as that of non-vaccinated mice on day 4 *p.i.*, before it increases again reaching maximal expression at peak parasitaemia on day 8 *p.i.,* which is about the same as that towards the end of the crisis phase on day 11 *p.i.* Secondly, the majority of erythroid genes reach their maximal expression levels already at peak parasitaemia on day 8 *p.i.* and keep about the same level during crisis, in contrast to non-vaccinated mice in which the corresponding genes reach their maximal expression levels on day 11 *p.i.* Thirdly, the final expression levels on day 11 *p.i.* are mostly lower with lower standard deviations in vaccination-protected mice than those of the corresponding genes in the liver of non-vaccinated un-protected mice (cf. Figs. 3, 4, 5, 6). A few genes in the liver of vaccinated mice, which still show a minor increase of mRNA expressions after peak parasitaemia on day 8 *p.i.*, slow down their expressions towards the end of the crisis phase on day 11 *p.i.* Some genes, as e.g. *Rhag*, *Epb4.1*, *Sptb*, *Tmod1* and *Acyp1*, have even significantly declined their expression levels on day 11 *p.i.*

Remarkably, the time course of expression of *Epor* in the liver of vaccinated mice is very similar to that of other genes proceeding from a level of about 25 on day 1 *p.i.* and reaches maximal levels of almost 100 on days 8 and 11 *p.i.* (Fig. 6). Also, the increase in expression of *Epor* between day 1 *p.i.* and day 4 *p.i.* as well as the expression on day 4 *p.i.* is significantly higher in vaccinated mice than in non-vaccinated mice. Remarkably, the time course of *Epor* expression significantly differs to that of *Epo*. Indeed, *Epo* expression is delayed during the first 4 days of infections, i.e., *Epo* is expressed at approximately the same very low level between day 0 *p.i.* and 4 *p.i.* in vaccinated mice as in non-vaccinated mice. Moreover, the maximal levels of *Epo* expression at peak parasitaemia on day 8 *p.i.* are significantly higher in the liver of vaccinated mice than in non-vaccinated mice (Fig. 6), though vaccination decreases maximum parasitaemia by approximately 30% in comparison with non-vaccinated mice [8, 43].

### Quantitative PCR validates the response of erythroid genes to infections

**Fig. 7:**
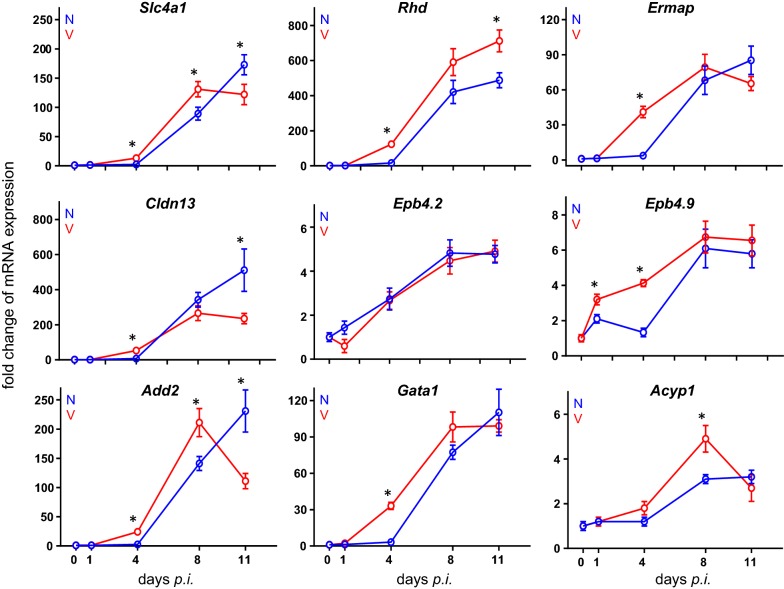
Quantitative PCR of mRNAs in the liver of vaccinated and non-vaccinated Balb/c mice. RNA was isolated from individual livers prepared from mice infected with *P. chabaudi* on different days *p.i.* Duplicate determinations were performed with liver aliquots from 3 different mice, and means are given ± SD. ‘*’ indicate significant differences between vaccinated and non-vaccinated mice (p < 0.05)

Quantitative PCR (qPCR) was used to re-examine, in both non-vaccinated and vaccinated mice, the time courses of expression of some arbitrarily selected erythroid genes, whose expression was identified by microarrays to respond to *P. chabaudi* blood-stage malaria. The data are summarized in Fig. 7. The trajectories of *Ermap* expression in both vaccinated and non-vaccinated mice during infection take a similar course as those detected by the microarray probes (Fig. 3). Moreover, the qPCR-determined trajectories for expression of *Gata1*, *Add2*, *Slc4a1*, *Rhd*, and *Cldn13* take a similar course as those determined by microarrays (cf. Fig. 7 with Figs. 3, 4, 5). In particular, there a higher increase in expression was observed at early patency on day 4 *p.i.* and a lower expression towards the end of the crisis phase on day 11 *p.i.* in vaccinated mice than in non-vaccinated mice. Furthermore, correspondence largely exists between microarray and qPCR data with respect to the tendency of increasing expression for *Epb4.2, Epb4.9*, and *Acyp1* (Fig. 7). Similarly to microarray data, the 9 genes examined by qPCR reveal maximal expression levels in the liver of vaccinated mice at peak parasitaemia on day 8 *p.i.*, whereas, in the liver of non-vaccinated mice, maximal expression of the majority of these genes is reached towards the end of crisis phase on day 11 *p.i.* Furthermore, the expressions of *Epor* and *Epo* measured by qPCR take very similar courses in vaccinated and non-vaccinated mice as those determined by microarrays (Fig. 6).

## Discussion

This study provides evidence that vaccination, using a non-infectious vaccine consisting of erythrocyte ghosts isolated from *P. chabaudi*-infected erythrocytes, accelerates expression of erythroid genes in the liver induced by blood-stage malaria of *P. chabaudi*. Indeed, these genes encompass *Slc4a1* and *Gypa* encoding band 3 and Glycophorin A, major integral membrane proteins of red blood cells; *Rhd*, *Kel* and *Rhag* coding for blood group antigens and Rh-associated antigen glycoprotein exposed on the outer membrane surface of red blood cells; *Ermap* and *Cldn1* encoding presumably proteins associated with the outer surface of erythroblasts [25]; *Add2* and *Ank1* coding for proteins linking integral membrane proteins with peripheral skeletal proteins located on the inner surface of erythroid cells, as e.g., *Epb4.2*, *Epb4.9*, *Spta,* and *Sptb* [45, 46]; *Acyp1* and *Ahsp* encoding proteins within erythroid cells; and even *Gata1*, *Gfi1b*, *Tal1* and *Klf1* coding for transcription factors critically involved in erythropoiesis [47–49]. Expression of these genes takes an accelerated time-course in surviving vaccinated mice, evidenced as it is significantly higher at early patency on day 4 *p.i.* and as it is often more slowed down or even lower towards the end of the crisis phase on day 11 *p.i.* than those expression time-courses taken by the corresponding genes during lethal infections in non-vaccinated mice. Thus, the results indicate that the liver responds to blood-stage malaria with extramedullary erythropoiesis, especially erythroblastosis in the liver, and erythroblastosis is obviously accelerated by vaccination.

Erythroblastosis is critically dependent on the interplay of EPO and EPOR, respectively [23, 24]. In accordance, extramedullary erythroblasts in the liver apparently display EPO sensitivity, evidenced as *Epor* expression taking approximately the same time-course as that taken by the other erythroid genes in vaccination-protected mice. Moreover, it is remarkable that *Epo* expression also occurs in the liver during extramedullary erythroblastosis. Indeed, there is evidence that EPO is not only produced by kidneys, but also by other cells including hepatocytes [23, 50]. In contrast to *Epor* and the other examined erythroid genes, however, hepatic expression of *Epo* is not accelerated during extramedullary erythroblastosis, rather it is delayed at least for the first 4 days of infections. Maximal *Epo* expression is reached at peak parasitaemia on day 8 *p.i.* both in vaccinated and non-vaccinated mice, but is much higher in vaccinated mice than in non-vaccinated mice, though, incidentally, non-vaccinated mice exhibit a higher peak parasitaemia by about 30% than vaccination-protected mice. The data, therefore, suggest that EPOR-mediated EPO sensitivity by hepatic erythroblasts precedes EPO production in the liver, and that malaria-induced erythroblastosis in the liver may require hepatic EPO particularly at peak parasitaemia. However, it cannot be excluded that hepatic EPO may be also involved in the stimulation of other processes in the liver, which are important to survive blood-stage malaria. For instance, EPO has been recently described to recruit Ly6C^hi^ monocytes in response to liver injury [51] and to stimulate proliferation and phagocytotic activity of Kupffer cells—the liver-resident macrophages known for its capacity of erythrophagocytosing *Plasmodium*-parasitized red blood cells [11, 12, 14–16]. Liver injury does also occur during blood-stage malaria in mice and even humans [11, 52]. Moreover, EPO has been reported to inhibit inflammation and gluconeogenesis in the liver [53]. Attenuated inflammation of the liver and decreased glucose blood levels after crisis was also found in vaccination-protected mice infected with *P. chabaudi* [8, 11].

Extramedullary erythroblastosis in the liver of vaccination-protected mice may be reasonably assumed to contribute to overcome malaria-induced anemia and replenishment of peripheral erythrocytes, whose number is increasingly declining with progressing *P. chabaudi* blood-stage infections. This view is supported by previous results obtained under identical experimental conditions as used here [8]. Indeed, *P. chabaudi*-parasitized erythrocytes begin to appear in peripheral blood of both vaccinated and non-vaccinated mice on day 4 *p.i.*, and peripheral red blood cells in both vaccinated and non-vaccinated mice begin to decrease after day 5 *p.i.*, *i.e.*, from approximately 9 × 10^6^/µl on day 5 *p.i.* to approximately 5.5 × 10^6^/µl on day 6 *p.i.* The lowest levels of approximately 1.8 × 10^6^/µl peripheral red blood cells were reached at peak parasitaemia on day 8 *p.i.* [8]. This very low level of peripheral erythrocytes remains in non-vaccinated mice until deceasing at latest at the end of the crisis phase on approximately day 11 *p.i.*, though there is observable a slight increase of peripheral reticulocytes at that time. In vaccinated mice, however, the increase in peripheral reticulocytes is accelerated and already begins about 1–2 days earlier than in non-vaccinated mice reaching higher levels at the end of crisis on day 11 *p.i.* [8]. This earlier appearance of peripheral reticulocytes may be due to an accelerated terminal erythroid differentiation in vaccination-protected mice, *i.e.*, final denucleation of erythroblasts and release of denucleated reticulocytes into liver sinusoids and blood vessels, respectively, begin 1–2 days earlier in vaccination-protected mice than in unprotected non-vaccinated mice.

The accelerated extramedullary erythropoiesis in the liver of vaccination-protected mice may be also viewed as an important contribution of the liver to the host defense system against blood-stage malaria. At the end of crisis on day 11 *p.i.*, for example, when parasitaemia has dramatically declined to about 1–5% in both vaccinated and non-vaccinated mice, there is accumulated a large excess of peripheral reticulocytes, representing approximately 90% of all peripheral red blood cells, only in vaccination protected mice [8]. This suggests that, at least at that time-point, vaccinated mice are less vulnerable to blood-stage infections, since reticulocytes—in contrast to erythrocytes—are not preferred as host cells by *P. chabaudi* [54]. A major reason for the host cell specificity of *P. chabaudi* for erythrocytes may be that reticulocytes differ from erythrocytes with respect to composition, organization and properties of their plasma membranes [45, 46]. Even erythrocytes during their aging are subjected to modifications in membrane components. For instance, there is evidence for an increase in the ratio of band 4.1a:4.1b during erythrocyte aging [55]. These hub proteins are not only involved in the organizational cross-linking of the spectrin network to the inner surface of red cell plasma membranes [56], but also favour the formation of elliptotic red cells, which are known to hinder red cell invasion by *Plasmodium* parasites [57, 58]. In accordance, there has been previously described a decrease of band 4.1a in erythrocytes of C57BL/10 mice after survival of *P. chabaudi* blood-stage infections [59]. Remarkably, the present study shows that the time course of *Epb4.1* expression differs from that of all the other examined erythroid genes insofar as there is dramatic decline on day 4 *p.i.*, when all other erythroid genes reveal an increased expression. Furthermore, there is ample evidence for other abnormalities, deficiencies and/or mutations of erythrocytes in their membrane or internal constituents which restrict invasion and/or internal survival of malaria parasites, respectively [60, 61]. It is therefore attractive to speculate that the accelerated extramedullary erythroblastosis in the liver of vaccination-protected mice ultimately leads, by the end of crisis phase, to an earlier transient excess of peripheral reticulocytes, being unfavourable for parasite survival, thus resulting in a dramatic decline of potential erythrocyte host cells for *P. chabaudi* parasites. This temporary impairment of parasite multiplication may concomitantly allow the evolvement of immune mechanisms finally contributing to resolve blood-stage infections of *P. chabaudi*.

## Conclusion

This study indicates that vaccination accelerates extramedullary erythroblastosis, induced in the liver by blood-stage malaria of *P. chabaudi,* for 1–2 days. This presumably contributes to earlier replenishment of peripheral red blood cells by liver-derived reticulocytes. This in turn is speculated to favour final survival of otherwise lethal blood-stage malaria, since reticulocytes—in contrast to mature erythrocytes—are known to be not preferred as host cells by *P. chabaudi*.

## Data Availability

The datasets used and/or analysed during the current study are available from the corresponding author on reasonable request. The microarray data have been deposited at the NCBI’s Gene Expression Omnibus (GEO) database with accession number GSE129133.

## Abbreviations

ACYP1: acylphosphatase 1 erythrocyte isozyme
ADD2: adducin2
AHSP: α haemoglobin stabilizing protein
ANK1: ankyrin1
CLDN13: claudin13
CFU-e: colony-forming unit-erythroid
EPB4.1: Erythrocyte membrane protein band 4.1
EPB4.2: Erythrocyte membrane protein 4.2
EPB4.9: Erythrocyte membrane protein 4.9
EPO: erythropoietin
EPOR: erythropoietin receptor
ERMAP: erythroblast membrane associated protein
FCA: Freund’s complete adjuvant
GAPDH: glycerinaldehyd-3-phosphat-Dehydrogenase
GATA1: GATA-binding factor 1
GFI1B: growth factor independent 1B transcriptional repressor
GYPA: glycophorin A
KEL: Kell blood group antigen
KLF1: Krueppel-like factor 1
PCA: principal component analysis
RHAG: Rh-associated glycoprotein
RHD: Rh blood group D antigen
SLC4A1: solute carrier family 4 member 1
SPTA1: spectrin α erythrocytic 1
SPTB: spectrin β erythrocytic
TAL1: T cell acute lymphocytic leukemia protein 1
TMOD1: tropomodulin 1
